# Inflammation Indices as Predictive Markers of Muscle-Invasive Bladder Cancer

**DOI:** 10.3390/cancers18010136

**Published:** 2025-12-31

**Authors:** Maciej Jaromin, Piotr Kutwin, Tomasz Konecki, Dariusz Popiela, Mateusz Kamecki, Marcin Kurowski

**Affiliations:** 1 1st Urology Department, Medical University of Lodz, 90-419 Łódź, Poland; 2 Department of Chemotherapy, Medical University of Lodz, 90-419 Łódź, Poland; 3 Department of Immunology and Allergy, Medical University of Lodz, 90-419 Łódź, Poland; marcin.kurowski@umed.lodz.pl

**Keywords:** bladder cancer, SII, SIRI, PLR, PIV, inflammation index, inflammation indices, diagnostics, prediction model, NMICB, MICB

## Abstract

Introduction: Prompt diagnosis and differentiation between non-muscle-invasive bladder cancer (NMIBC) and muscle-invasive bladder cancer (MIBC) are essential in the treatment of bladder cancer. Inflammation-based biomarkers have recently emerged as potential tools for improving cancer diagnostics and prognostication. This study aims to evaluate the potential value of the Systemic Inflammation Index (SII), Systemic Inflammation Response Index (SIRI), Pan-immune Inflammation Value (PIV), and Platelet-to-Lymphocyte Ratio (PLR) as predictors of muscle-invasive disease. Materials and methods: Analyzed data included 310 bladder tumors. The SII, SIRI, PIV, and PLR were calculated from pre-TURBT complete blood-count results. Differences in inflammatory markers between pathological stages (pTa–pT4) were examined using ANOVA with Tukey’s post hoc testing. Optimal cutoff values for distinguishing NMIBC from MIBC were identified using ROC curve analysis and Youden’s J statistic. Logistic regression models incorporating age, sex, the number of recurrences, and each inflammatory index were developed to evaluate their predictive performance in patients treated with curative intent. Results: All investigated inflammation indices significantly differed across tumor stages (*p* < 0.001), with lower values observed in pTa tumors compared with muscle-invasive disease. Determined cutoff values for muscle-invasive disease were 865.63 for SII, 2.02 for SIRI, 579.28 for PIV, and 166.35 for PLR. Logistic regression models demonstrated promising diagnostic performance, achieving AUC values of 0.812 (SII), 0.816 (SIRI), 0.821 (PIV), and 0.795 (PLR); sensitivity and specificity were 76% and 75% for SII, 79% and 77% for SIRI, 80% and 72% for PIV, and 88% and 59% for PLR. Discussion: The presented results indicate that inflammation-based indices vary meaningfully between bladder cancer stages and may be utilized in early identification of muscle-invasive disease. As inexpensive and widely available biomarkers, they offer potential value in the evaluation of suspected MIBC before the final pathology report and could enhance the diagnostic process.

## Simple Summary

Bladder cancer treatment depends strongly on whether the tumor has invaded the muscle layer (muscle-invasive) or not, making early and accurate distinction crucial. This study explored whether inflammation markers calculated from complete blood-count results could help identify muscle-invasive disease before definitive pathology is available. We evaluated four inflammation-related indices—the Systemic Inflammation Index (SII), Systemic Inflammation Response Index (SIRI), Pan-immune Inflammation Value (PIV), and Platelet-to-Lymphocyte Ratio (PLR)—calculated from pre-TURBT blood tests in 277 patients with bladder cancer. All indices showed clear differences across tumor stages, with higher values associated with more advanced, muscle-invasive disease. In logistic regression analysis, each index demonstrated good diagnostic accuracy for prediction of muscle invasion, with acceptable sensitivity and specificity. Since these indices are inexpensive, widely available, and easy to calculate, they may support clinicians in early risk stratification and decision-making, potentially improving the diagnostic pathway for patients with suspected muscle-invasive bladder cancer.

## 1. Introduction

Bladder cancer (BC) is linked to significant morbidity and mortality, and it imposes one of the highest economic costs per person among all cancer types because of requirements for intensive treatment and ongoing lifelong monitoring. At initial presentation, 70–75% of patients have non-muscle-invasive bladder cancer (NMIBC), 20–25% have muscle-invasive bladder cancer (MIBC), and 5% have metastatic disease [1,2,3]. BC is the ninth most common malignancy globally and shows increasing incidence trends, with approximately 614,298 new cases diagnosed worldwide in 2022, representing a 7.1% increase from 2020 [4,5]. In Poland, the overall cancer mortality rate was 283 deaths per 100,000 population in 2019, exceeding the European Union average by 15%. Despite Poland’s generally lower overall cancer incidence rates compared to the EU average, the notably higher mortality rates indicate potential delays in diagnosis and challenges related to timely access to or quality of healthcare services. Additionally, mortality from bladder cancer increased in Poland between 2011 and 2019 by approximately 8% [6,7].

Diagnosis usually starts after painless macroscopic hematuria appears. Tissue samples, confirming cancer and determining the stage, are obtained through transurethral resection of a bladder tumor (TURBT), which serves both diagnostic and therapeutic purposes [8]. Various guidelines are available with respect to the management of bladder cancer, including those from the European Association of Urology, the American Urological Association, and the Society of Urologic Oncology. In general, NMIBCs are frequently managed with endoscopic resection and eventual BCG intravesical therapy, depending on progression risk. In contrast, MIBCs are managed with more aggressive treatments such as cystectomy (bladder removal) with or without neoadjuvant chemotherapy [9,10].

Recent years have seen the emergence of systemic inflammation indices derived from routine blood tests as potential biomarkers of disease course or treatment-response predictors across various malignancies [11,12], as well as cardiovascular [13], metabolic [14], respiratory [15], and allergic [16] conditions. Pre-operative complete blood-count (CBC) tests are standard procedures for almost all admitted patients. Common indicators in blood tests include white blood cell count, neutrophil count, monocyte count, lymphocyte count, platelet count, and albumin concentration—certain inflammatory indices can be derived through straightforward calculations of these indicators. Examples of these inflammation indices include the Systemic Inflammation Index (SII), Systemic Inflammation Response Index (SIRI), Pan-immune Inflammation Value (PIV), and Platelet-to-Lymphocyte Ratio (PLR). These may serve as potential biomarkers to complement traditional medical pathology and help tailor clinical treatment strategies for bladder cancer patients, but more evidence is needed [17].

Considering these facts, we set out to assess possible differences in the values of selected systemic inflammation indices (SII, SIRI, PIV, and PLR) in bladder cancer patients depending on cancer stage. In addition, our goal was to assess and analyze the potential utility of the SII, SIRI, PIV, and PLR as predictors of muscle-invasive disease.

## 2. Materials and Methods

This study evaluate data retrospectively acquired from the databases of urology departments at two university hospitals (2nd Clinical Hospital and Copernicus Memorial Hospital) in Lodz, Poland. The suspected tumors were detected in flexible cystoscopy and/or radiological imaging prior to hospitalization. Patients included in the study were treated for urothelial bladder cancer in different stages; the analysis included both patients treated with curative intent and patients in palliative care. Data collection was anonymous, did not require patient involvement, and did not alter any medical or therapeutic decisions. The following exclusion criteria were employed: (1) concomitant oncological, hematological, or rheumatological conditions; (2) symptoms of active infection (including urinary infections); (3) inborn or acquired immunodeficiency; and (4) any other condition that might possibly influence white blood-cell count and proportions between immune system cells, as judged and agreed upon by the investigators. Any antiplatelet or anticoagulant medications were laid off at least 4 days before the procedure, with exclusion of subcutaneous enoxaparin in a standard prophylactic dose.

Importantly, data was acquired from the CBC before patients’ first TURBT procedure, before the histopathology report was known. If the tumor was recurrent and did not progress, the subsequent morphologies were not taken into consideration. If the tumor progressed, corresponding tumors and CBC results were included in the analysis, e.g., a pT1 tumor that recurred and progressed to a pT2 tumor has two corresponding morphologies: one taken before detection of the pT1 tumor and second taken before detection of the pT2 tumor.

The following formulas were used to calculate inflammation indices:

1. Systemic Inflammation Index (SII):
  SII = (Platelets × Neutrophils)/Lymphocytes;
2. Systemic Inflammation Response Index (SIRI):
  SIRI = (Neutrophils × Monocytes)/Lymphocytes;
3. Pan-immune Inflammation Value (PIV):
  PIV = (Neutrophils × Platelets × Monocytes)/Lymphocytes;
4. Platelet-to-Lymphocyte Ratio (PLR):
  PLR = Platelets/Lymphocytes

The statistical analysis was performed in RStudio version 2025.09.0 + 387 for Windows. Utilized packages, all accessible via https://cran.r-project.org/web/packages/available_packages_by_name.html (accessed on 26 December 2025) website, included “plotly”, “readxl”, “corrplot”, “parameters”, “gplot2”, “sm”, “dplyr”, “MASS”, “multcomp”, “lsr”, “pROC”, “caret”, “cluster”, and “scales” [18,19,20,21,22,23,24].

Data was tested for normality with the Shapiro–Wilk test, and normal distribution was rejected in cases of *p*-values < 0.05. Data with a normal distribution was compared with a *t*-test, while data with a non-normal distribution was compared with Wilcoxon’s rank-sum test.

An ANOVA was performed on data normalized by the Box–Cox transformation. The post hoc analysis was performed using Tukey’s Honestly Significant Difference (HSD) test. The binary logistic regression model was developed using the “glm()” function. Variables including age, SII, SIRI, PIV, and PLR were normalized using the “scale()” function. The optimal cutoff threshold for the logistic regression model was calculated using Youden’s J statistic.

## 3. Results

### 3.1. Characteristics of the Study Group

The study group included 277 patients diagnosed with different stages (CIS/pTa/pT1/pT2/pT3/pT4) and grades (LG/HG) of bladder cancer. Full characteristics of the study group are presented in Table 1. Male patients (n = 198) contributed to 71.5% of the study group; the mean age of male patients was 68 years, and the mean age of female patients was 70 years. In-patients primarily treated with curative intent (n = 222) contributed to 80.1% of the study group, while 11 patients progressed to palliative disease and n = 55 patients (19.9%) were palliative at first diagnosis.

It is important to note that during follow-up, 33 patients progressed and had tumors of different stages, so the number of analyzed tumors (n = 310) is higher than the number of patients. Carcinoma in situ (CIS) was present in 8 patients: 6 with pT1 tumors and 2 with pTa tumors.

### 3.2. ANOVA Tests

The values of inflammation indices differentiated by tumor stage are presented in Table 2.

**Figure 1:**
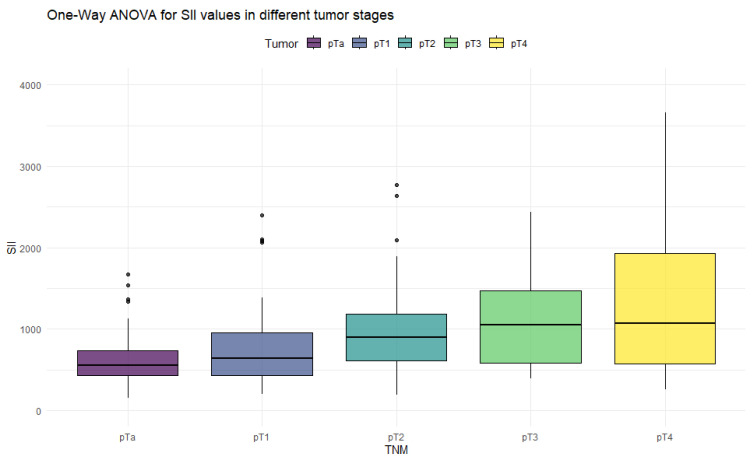
Distribution of SII values in different stages of bladder cancer.

**Figure 2:**
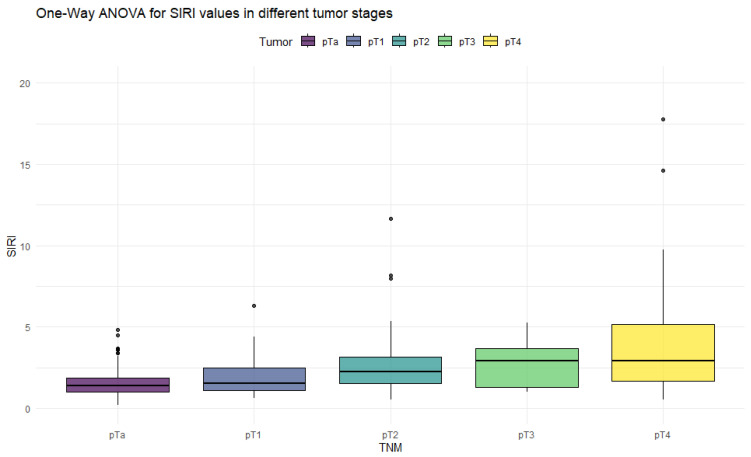
Distribution of SIRI values in different stages of bladder cancer.

**Figure 3:**
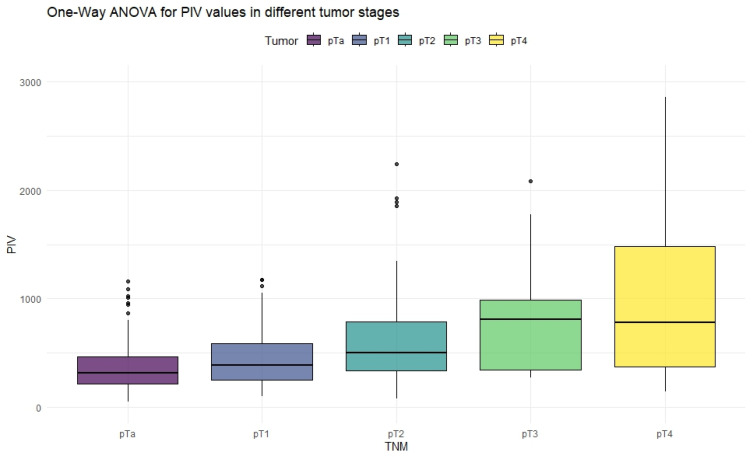
Distribution of PIV values in different stages of bladder cancer.

**Figure 4:**
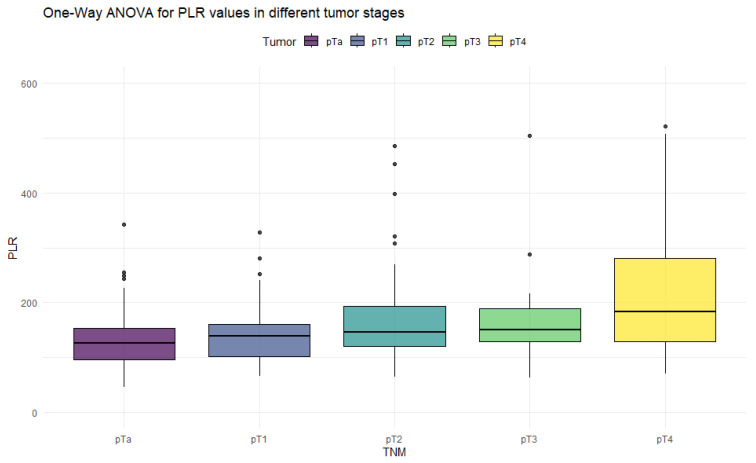
Distribution of PLR values in different stages of bladder cancer.

The ANOVA test with Box–Cox transformation was used to measure differences in values across different stages of bladder cancer. Figure 1, Figure 2, Figure 3 and Figure 4 provide a visual representation of the distribution of inflammation index values, and the ANOVA test results with post hoc analysis are presented in Table 3.

The ANOVA test showed significant differences in all inflammation indices, depending on the tumor stage. Patients with non-muscle-invasive bladder cancer had clearly lower values of inflammation indices than patients with muscle-invasive disease. PIV was the most differentiating factor, with the highest F values, reaching statistically significance in five tumor-stage comparisons. Importantly, only the PIV differed significantly between pT2 and pT4 tumors. Furthermore, *p*-values tended to be the lowest when comparing tumor stages using the PIV—even in non-significant comparisons.

### 3.3. Binary Logistic Regression

In the next part of analysis, we created a binary logistic regression model to check for the potential value of inflammation indices calculated from pre-TURBT CBC in predicting further therapeutic procedures.

The dichotomy of therapeutic approaches allows for a division of patients in two very distinct groups (**pTa + pT1** vs. **pT2 + pT3 + pT4**), characterized by the type of curative treatment they receive after initial resection. The inflammation index values for both groups are presented in Table 4. Since the model is designed to predict curative treatment in bladder cancer patients, the third group, i.e., patients qualified for palliative care, were excluded from this part of analysis.

To establish cutoff values of inflammation indices, an ROC curve with Youden’s J statistic was calculated. The continuous variables were SII, SIRI, PIV, and PLRT; the binary variable was assignment to a non-muscle-invasive group (pTa + pT1) vs. a muscle-invasive group (pT2+). The results are presented in Table 5.

**Figure 5:**
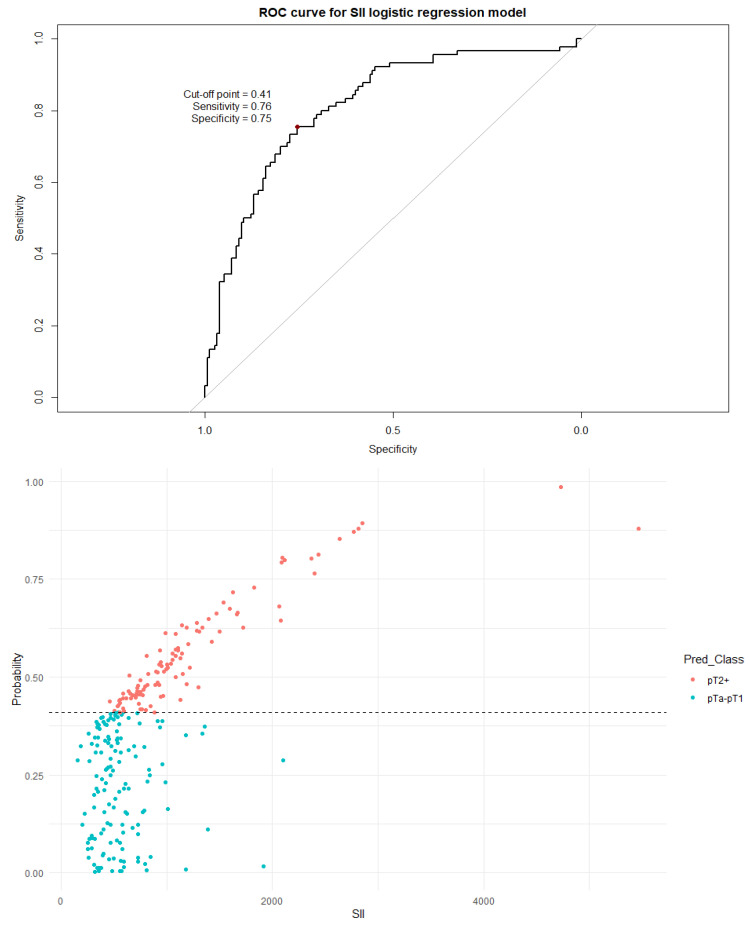
The ROC curve and distribution of analyzed data in binary logistic regression model including SII values. The optimal cutoff calculated via Youden’s J statistic was determined to be 0.41. AUC = 0.812; sensitivity = 76%; specificity = 75%. Impact of independent variables on the model: *p* = 0.50 for age, *p* = 0.56 for sex, and *p* < 0.001 for both the SII value and the number of recurrences.

**Figure 6:**
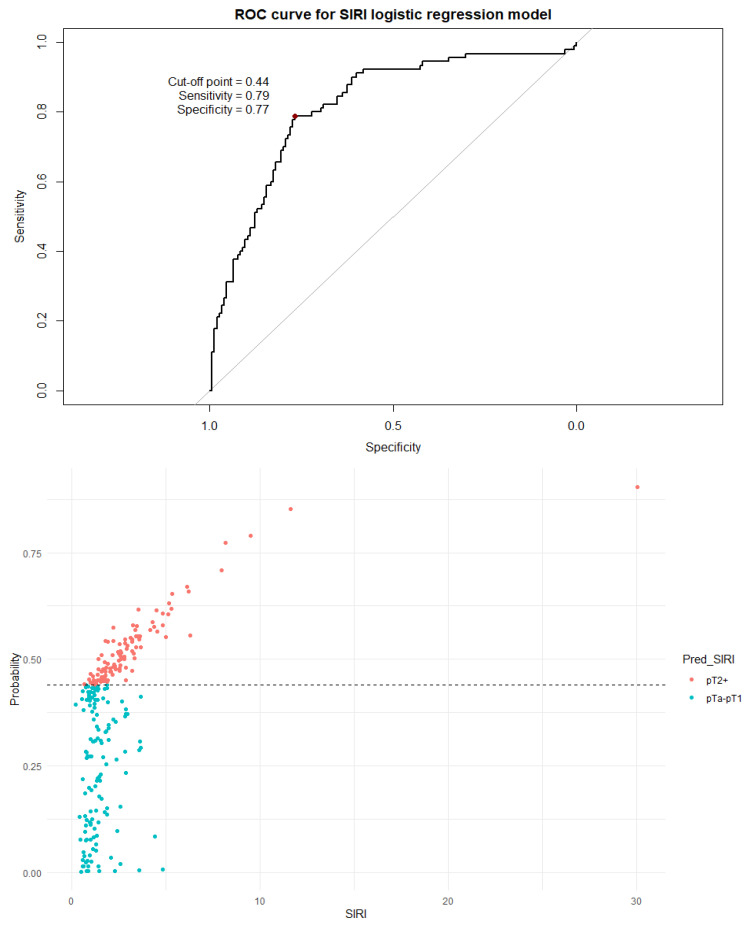
The ROC curve and distribution of analyzed data in binary logistic regression model including SIRI values. The optimal cutoff calculated via Youden’s J statistic was determined to be 0.44. AUC = 0.816; sensitivity = 79%; specificity = 77%. Impact of independent variables on the model: *p* = 0.46 for age, *p* = 0.87 for sex, *p* = 0.01 for SIRI value, and *p* < 0.001 for the number of recurrences.

**Figure 7:**
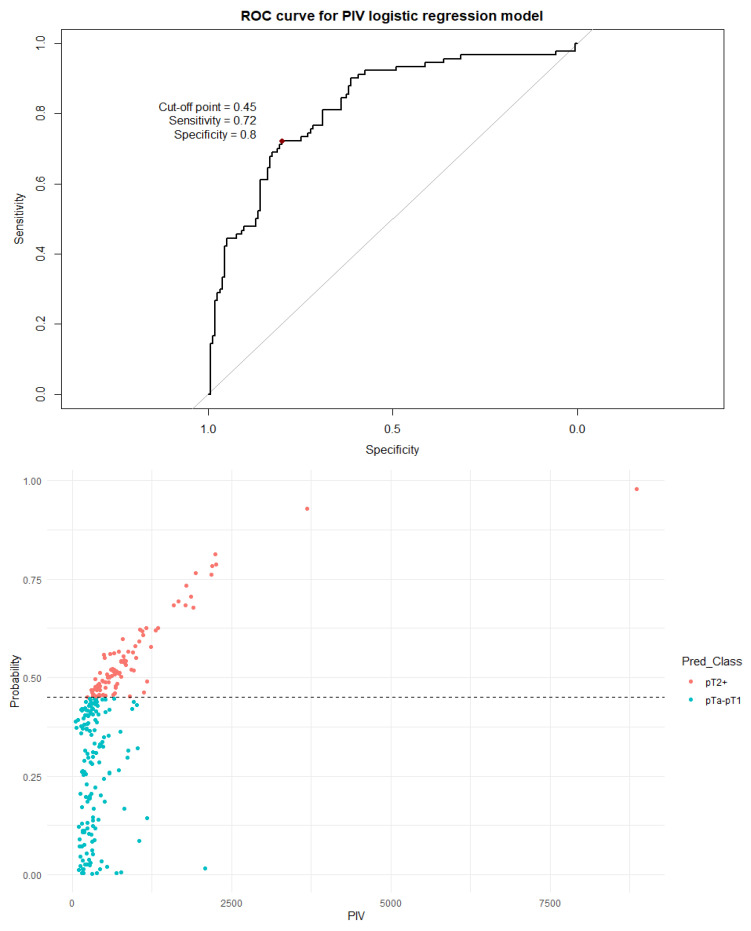
The ROC curve and distribution of analyzed data in binary logistic regression model including PIV values. AUC = 0.821; sensitivity = 72%; specificity = 80%. The optimal cutoff calculated via Youden’s J statistic was determined to be 0.45. Impact of independent variables on the model: *p* = 0.50 for age, *p* = 0.70 for sex, *p* = 0.008 for the PIV value, and *p* < 0.001 for the number of recurrences.

**Figure 8:**
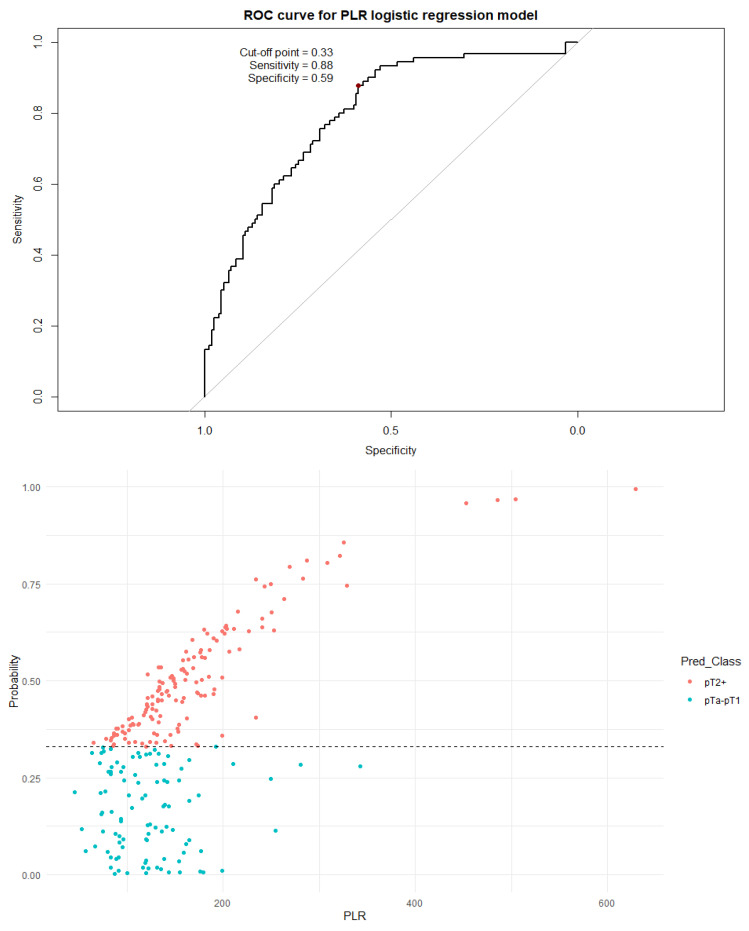
The ROC curve and distribution of analyzed data in binary logistic regression model including PLR values. The optimal cutoff calculated via Youden’s J statistic was determined to be 0.33 AUC = 0.795; sensitivity = 88%; specificity = 59%. Impact of independent variables on the model: *p* = 0.46 for age, *p* = 0.40 for sex, and *p* < 0.001 for both the PLR value and the number of recurrences.

Next, a binary logistic regression model was built for each individual index. The dependent variable was assignment to a non-muscle-invasive group (pTa or pT1) or to a muscle-invasive group (pT2+). The dependent variables included age, sex, the number of recurrences, and an inflammation index (SII, SIRI, PIV, or PLR). A visualization of the logistic regression predictive value, the ROC curve, and the distribution graph are presented in Figure 5, Figure 6, Figure 7 and Figure 8.

Every inflammation index used in this study had an impact on the regression model, with PLR and SII exerting the greatest influence (both *p* < 0.001). The number of recurrences had high predictive value in every model, most likely due to the highest number of recurrences occurring in the pTa group, in contrast with no recurrences in primarily palliative patients and patients with muscle-invasive disease detected after the first TURBT.

## 4. Discussion

The aim of the presented analysis was to assess the differences in inflammation indices in different stages of bladder cancer and their potential value in diagnostic and therapeutic processes. Analyzed tumors ranged from pTa LG to pT4 HG; however, the distribution of tumors used in this study (Table 2) is different from tumor distributions in typical patient populations [25,26]. The choice to over-represent muscle-invasive disease in the study group was dictated mostly by two factors—firstly, the volume of sample sizes should be comparable for viable statistical analysis, and secondly, diagnosis of muscle-invasive disease is more urgent than diagnosis of non-muscle-invasive bladder cancer.

The study focused on researching four inflammation indices: the Systemic Inflammation Index, Systemic Inflammation Response Index, Pan-immune Inflammation Value, and Platelet-to-Lymphocyte Ratio. The important differentiating factor between these indices is the involvement of the platelet count. Gross hematuria occurs commonly in patients with bladder cancer [27], and cancer cells have the potential to promote platelet aggregation via many different mechanisms [28,29]. Considering these factors, the platelet count could potentially distort the results, and the SIRI and PLR values may be less credible than the SII and PIV. This was not the case in ANOVA tests—PIV and PLR performed almost uniformly relative to the SII and SIRI, but in logistic regression, the PLR model had meaningfully lower specificity than the PIV, SIRI, and PIV models.

The ANOVA part of the analysis showed significant differences in values of the inflammation indices depending on tumor stage. The highest disparities were shown in comparison between pTa/pT1 and pT4 tumors; moreover, every inflammation index differed between pTa tumors and muscle-invasive tumors. The differences between pT1 tumors and muscle-invasive tumors were less distinct—metanalysis or studies with greater sample sizes should be conducted to clarify whether these observations are, indeed, statistically significant. Two metanalyses proved that SII values are reliable in differentiating <pT3 tumors from ≥pT3 tumors [30,31], but we were unable to find a study reporting a comprehensive comparison of all bladder cancer stages.

It should be noted that all indices differed significantly (*p* < 0.001) between non-muscle-invasive and muscle-invasive bladder cancer patients, even after including only localized disease (Table 5). The cutoff values established by the ROC curve and Youden’s J statistic resulted in relatively high specificity but low sensitivity and modest AUC values. To better utilize the inflammation indices in a clinical setting, we decided to develop a more complex binary logistic regression model. The model included age, sex, the number of recurrences, and a single inflammation index as independent variables; the dependent variable was the distinction between NMIBC and MIBC. The independent variables were chosen as information easily obtainable before every TURBT procedure. Tests including the SII, SIRI, and PIV were very similar in strength, with approximately 80–76% sensitivity and 77–72% specificity. The model including PLR as an independent variable was clearly subpar compared to other models, with a very low cutoff threshold of 0.33 and a quite low specificity of 59%. There is no doubt that values of inflammation indices are not as impactful on the type of proposed treatment as a histopathology report, but initial assessment based on pre-TURBT CBC and the patient’s history may be influential in the beginning of the diagnostic process.

This study has some limitations. Firstly, the data was collected retrospectively, which narrowed available data and prevented additional measurements. The study group was designed to heavily focus on muscle-invasive cases and not to mirror the actual distribution of stages and grades in bladder cancer patients. This may potentially limit the usefulness of the presented results in diagnosis of non-muscle-invasive disease. Furthermore, the study group of 277 patients was enough to provide meaningful results, but it was not enough for training and testing of the regression model. In exchange, we performed cross-validation of regression models; the results of cross-validation of logistic regression models included in the study are presented in Supplementary File S1. The cross-validation results align with the presented AUC values, but the specificity and sensitivity are slightly lower. The most important limitation was probably the fact that some patients were lost to follow-up. A considerable portion of collected data comes from the period between 2019 and 2022, the time in which COVID-19 regulations reshaped and restructured many aspects of healthcare. The relatively short median follow-up time results from very poor prognosis in palliative patients, but in some patients, the follow-up was lost during the time of the COVID-19 pandemic, most likely due to patients switching outpatient clinics.

In recent years, the body of literature regarding the usefulness of inflammation indices in diagnosis of various cancers has constantly grown. Emerging metanalyses report that the values of inflammation indices correspond with prognoses such as overall survival and disease-free survival in breast cancer, prostate cancer, gastrointestinal cancers, and lung cancers, among others [32,33,34,35,36]. Elevated levels of the SII and SIRI are also associated with reduced response rates to immunotherapy, further suggesting its use as a predictor of therapeutic efficacy [37,38].

The literature regarding the utility of inflammation indices in bladder cancer is also rapidly developing. One metanalysis by Wei et al. assessed the prognostic value of the SII in predicting the overall survival, cancer-specific survival, progression-free survival, and recurrence-free survival in bladder cancer patients. The proposed cutoff value was SII = 507; the prognosis for all examined metrics was clearly unfavorable for patients with SII < 507 [39]. A recent paper by Bizzarri et al. retrospectively evaluated the prognostic value of inflammation indices in predicting recurrence-free survival in 285 bladder cancer patients. The cutoff values were calculated with the same method as in this study (ROC curve with Youden’s Index) and determined to be 327 for SII, 1.12 for SIRI, 248.48 for PIV, and 139 for PLR [40]. In comparison to our study, the thresholds presented in both abovementioned studies are lower than the calculated cutoff values for all inflammation indices. These disparities may result from different outcome measures, since our study focused on patient assignment to NMIBC or MIBC groups.

In bladder cancer, inflammation indices may be utilized to predict another very important therapeutic outcome—patients’ response to intravesical BCG instillations. A study by Akan et al. retrospectively evaluated pre-TURBT levels of the SII, PLR, and NLR in high-risk NMIBC patients. The group with complete response (n = 59) was compared to BCG-unresponsive (n = 16) and BCG-relapsing (n = 21) patients; in both cases, the SII was lower (450.5 vs. 793.3 and 450.5 vs. 686.6, respectively) in the complete response group, but PLR differed significantly only between BCG-responsive and BCG-unresponsive patients (113.2 vs. 146.7, *p* = 0.005) [41]. Similar findings were reported by Ye et al. in a study analyzing the role of SIRI and PLR levels (among other factors) with respect to positive response to BCG therapy. In multivariate analysis, high SIRI values (cutoff value = 0.785, *p* < 0.001) were reported to be an independent predictor of BCG response [42]. However, a study by Bolat et al. did not show any statistically significant differences in PLR and SII levels either in comparison between responsive vs. recurrent patients or in comparison between responsive vs. progressing patients [43]. A metanalysis combining data of 12,645 patients confirmed that SII values in BCG-responsive patients are lower than in BCG-unresponsive patients (standardized mean difference = −1.04) [30]. The usage of inflammation indices in predicting the effectiveness of BCG therapy is very promising; the inclusion of inflammation indices, especially the SII, in the therapeutic process may help in identifying patients with NMIBC not suited for BCG instillations and the precipitate potential of radical cystectomy.

Future studies may also focus on monitoring the results of immune checkpoint inhibitor (ICI) therapy in patients with muscle-invasive bladder cancer, as well as providing mechanistic insights into observed increases in inflammation indices. Hypothetically, a rise in inflammation indices may suggest the progression of the disease and help with prompt detection of disease progression, and different levels of inflammation indices may have prognostic value in the assessment of ICI efficacy and progression-free survival.

## 5. Conclusions

Inflammation indices differ between stages of bladder cancer, with a clear tendency to rise with advanced stages. The information provided by inflammation indices are swift and accessible and generate a close-to-negligible financial burden for the patient and the hospital. The inclusion of inflammation indices in bladder cancer diagnostics, especially initial differentiation between NMIBC and MIBC before the final histopathology report and assessment of responsiveness to BCG therapy, may help in the therapeutic process of the most challenging patients.

## Figures and Tables

**Table 1:** Characteristics of the study group.

	Study Group n = 277
Sex (male; female)	198; 79
Age	68.7 ± 8.7
Hypertension	126
Diabetes	47
Median follow-up (months)	26
Recurring tumors	104
Progressions	33
Deaths during follow-up	78
Received BCG instillations	38
Progression after BCG	7
Received neoadjuvant therapy	38
Hemoglobin (Hb) in males (g/dL)	13.15 ± 2.37
Hemoglobin in females (g/dL)	12.61 ± 1.68
Platelet count (×10^9^/L)	261.54 ± 84.36
White blood-cell count (×10^6^/L)	8.24 ± 2.50
Lymphocytes (×10^6^/L)	1.79 ± 0.66
Neutrophiles (×10^6^/L)	5.58 ± 2.29
Monocytes (×10^6^/L)	0.66 ± 0.24
Tumor grade (LG; HG)	82; 228
pTa Tumors	113
pT1 Tumors	46
pT2 Tumors	53
pT3 Tumors	17
pT4 Tumors	81
M0, N+	24
M+	66

The norms for the CBC parameters are Hb = 12.0–16.0 g/dL (females), Hb = 13.0–16.5 g/dL (males), PLT = 150–400 × 10^9^/L, WBC = 4.0–10.0 × 10^6^/L, LYM = 1.3–2.9 × 10^6^/L, NEU = 2.2–4.8 × 10^6^/L, and MON = 0.3–0.8 × 10^6^/L.

**Table 2:** Values of chosen inflammation indices in different stages of bladder cancer.

	pTa	pT1	pT2	pT3	pT4
SII (95%CI)	552.23 244.89–1406.03	638.2 261.30–2096.25	888.64 222.34–2585.89	1052.05 416.50–2309.54	1081.24 290.90–6024.93
SIRI (95%CI)	1.39 0.45–3.87	1.47 0.63–4.40	2.34 0.65–8.12	2.92 1.05–5.10	2.92 0.75–9.73
PIV (95%CI)	317.36 93.31–1039.14	387.57 121.45–1166.39	542.95 93.02–2155.91	809.57 274.07–1960.32	787.46 165.81–5620.95
PLR (95%CI)	124.20 56.0–230.58	138.51 72.02–276.07	146.48 67.45–437.99	158.55 79.67–579.25	184.83 85.21–521.43

**Table 3:** Results of ANOVA test and Tukey’s HSD post hoc test.

	SII	SIRI	PIV	PLR
Degrees of freedom	4	4	4	4
F value	17.16	15.79	19.92	13.16
Lambda (λ) in Box–Cox transformation	−0.34	−0.14	−0.22	−0.46
Shapiro–Wilk *p*-value	0.64	0.14	0.48	0.22
ANOVA *p*-value	<0.001	<0.001	<0.001	<0.001
Post hoc analysis	pTa–pT2 (*p* < 0.001) pTa–pT3 (*p* = 0.003) pTa–pT4 (*p* < 0.001) pT1–pT2 (*p* = 0.24) pT1–pT3 (*p* = 0.10) pT1–pT4 (*p* < 0.001)	pTa–pT2 (*p* < 0.001) pTa–pT3 (*p* = 0.015) pTa–pT4 (*p* < 0.001) pT1–pT2 (*p* = 0.11) pT1–pT3 (*p* = 0.19) pT1–pT4 (*p* < 0.001)	pTa–pT2 (*p* < 0.001) pTa–pT3 (*p* = 0.002) pTa–pT4 (*p* < 0.001) pT1–pT2 (*p* = 0.13) pT1–pT3 (*p* = 0.07) pT1–pT4 (*p* < 0.001) pT2–pT4 (*p* = 0.027)	pTa–pT2 (*p* = 0.007) pTa–pT3 (*p* = 0.039) pTa–pT4 (*p* < 0.001) pT1–pT2 (*p* = 0.20) pT1–pT3 (*p* = 0.21) pT1–pT4 (*p* < 0.001)

The normalization of the inflammation index values was checked by the Shapiro–Wilk test to ensure the reliability of the analysis results. All differences between muscle-invasive stages were not significant (*p* > 0.05).

**Table 4:** Comparison of inflammation indices in two groups revealed high statistical significance (*p* < 0.001) between non-muscle-invasive and muscle-invasive bladder cancer patients.

	pTa + pT1, n = 155	pT2+, n = 90	*p*-Value
SII	565.15	944.77	<0.001
SIRI	1.40	2.41	<0.001
PIV	330.07	594.99	<0.001
PLR	129.78	152.41	<0.001

**Table 5:** ROC curve parameters and cutoff values for NMIBC.

	AUC	Sensitivity	Specificity	Cutoff Value
SII	0.709	57%	81%	865.62
SIRI	0.689	61%	75%	2.02
PIV	0.700	52%	81%	579.28
PLR	0.655	46%	83%	166.35

## Data Availability

Data is contained within the article or Supplementary Material.

## Supplementary Materials

The following supporting information can be downloaded at: https://www.mdpi.com/article/10.3390/cancers18010136/s1.
