# Supplementary material for: Inflammation Indices as Predictive Markers of Muscle-Invasive Bladder Cancer
Source: Cancers (Basel). 2025 Dec 31;18(1):136. doi: 10.3390/cancers18010136 (PMC12784776; doi:10.3390/cancers18010136)
Supplement: Supplementary file 1 [file cancers-18-00136-s001.zip › cancers-4018532-supplementary.pdf]

Supplementary File S1

The results of cross-validation of ligistic regression models for different inflammatory indexes

All models were cross validated with cut-off points establish via Youden's J index, namely:
0.41 for SII
0.44 for SIRI
0.45 for PIV
0.33 for PLR

Custtom function used to compute performance matrix („summaryFunction") was developped to include aforementioned Youden's J values:

```
customSummary <- function(data, lev = NULL, model = NULL) {
  prob <- data[[ lev[2] ]]

  pred_class <- ifelse(prob > [Selected Youden's J index value], lev[2], lev[1])

  cm <- caret::confusionMatrix(
    factor(pred_class, levels = lev),
    factor(data$obs,  levels = lev),
    positive = "pT2."
  )

  auc_val <- pROC::auc(data$obs, prob)

  out <- c(
    AUC = as.numeric(auc_val),
    Sensitivity = cm$byClass["Sensitivity"],
    Specificity = cm$byClass["Specificity"])

  return(out)
}
```

Raw results obtained via „trainControl" and „train" functions from „pROC" and „caret" R studio packages are presented below.

For SII:

245 samples
4 predictor
2 classes: 'pT1.pTa', 'pT2.'

No pre-processing
Resampling: Cross-Validated (10 fold)
Summary of sample sizes: 221, 221, 221, 220, 220, 221, ...
Resampling results:

AUC	Sensitivity.Sensitivity	Specificity.Specificity
0.8078704	0.7333333	0.7225

For SIRI:

245 samples
4 predictor
2 classes: 'pT1.pTa', 'pT2.'

No pre-processing
Resampling: Cross-Validated (10 fold)
Summary of sample sizes: 220, 220, 221, 220, 220, 221, ...
Resampling results:

AUC	Sensitivity.Sensitivity	Specificity.Specificity
0.7916667	0.7555556	0.7154167

For PIV:

245 samples
4 predictor
2 classes: 'pT1.pTa', 'pT2.'

No pre-processing
Resampling: Cross-Validated (10 fold)
Summary of sample sizes: 221, 220, 220, 220, 220, 221, ...
Resampling results:

AUC	Sensitivity.Sensitivity	Specificity.Specificity
0.8017593	0.6555556	0.7666667

For PLR:

245 samples
4 predictor
2 classes: 'pT1.pTa', 'pT2.'

No pre-processing
Resampling: Cross-Validated (10 fold)
Summary of sample sizes: 221, 221, 220, 221, 221, 220, ...
Resampling results:

AUC	Sensitivity.Sensitivity	Specificity.Specificity
0.7854167	0.8222222	0.5741667
